# Dicholine succinate, the neuronal insulin sensitizer, normalizes behavior, REM sleep, hippocampal pGSK3 beta and mRNAs of NMDA receptor subunits in mouse models of depression

**DOI:** 10.3389/fnbeh.2015.00037

**Published:** 2015-02-26

**Authors:** Brandon H. Cline, Joao P. Costa-Nunes, Raymond Cespuglio, Natalyia Markova, Ana I. Santos, Yury V. Bukhman, Aslan Kubatiev, Harry W. M. Steinbusch, Klaus-Peter Lesch, Tatyana Strekalova

**Affiliations:** ^1^Faculté de Médecine, INSERM U1119, Fédération de Médecine Translationnelle de Strasbourg, Université de StrasbourgStrasbourg, France; ^2^Department of Neuroscience, Maastricht UniversityMaastricht, Netherlands; ^3^Group of Behavioural Neuroscience and Pharmacology, Institute for Hygiene and Tropical Medicine, New University of LisbonLisbon, Portugal; ^4^Faculty of Medicine, Neuroscience Research Center of Lyon, INSERM U1028, C. Bernard UniversityLyon, France; ^5^Laboratory of Biomolecular Screening, Institute of Physiologically Active Compounds, Russian Academy of SciencesMoscow, Russia; ^6^Laboratory of Cognitive Dysfunctions, Institute of General Pathology and Pathophysiology, Russian Academy of Medical SciencesMoscow, Russia; ^7^Faculdade de Ciências Médicas, NOVA Medical School, Universidade Nova de LisboaLisboa, Portugal; ^8^Great Lakes Bioenergy Research Center, Computational Biology, Wisconsin Energy Institute, University of WisconsinMadison, WI, USA; ^9^Laboratory of Translational Neuroscience, Division of Molecular Psychiatry, Centre of Mental Health, University of WuerzburgWuerzburg, Germany

**Keywords:** chronic stress, insulin receptor, dicholine succinate, phosphorylated glycogen synthase kinase-3beta (pGSK-3beta), NMDA receptor subunits NR2A and NR2B, sleep EEG, aging, hippocampal plasticity

## Abstract

Central insulin receptor-mediated signaling is attracting the growing attention of researchers because of rapidly accumulating evidence implicating it in the mechanisms of plasticity, stress response, and neuropsychiatric disorders including depression. Dicholine succinate (DS), a mitochondrial complex II substrate, was shown to enhance insulin-receptor mediated signaling in neurons and is regarded as a sensitizer of the neuronal insulin receptor. Compounds enhancing neuronal insulin receptor-mediated transmission exert an antidepressant-like effect in several pre-clinical paradigms of depression; similarly, such properties for DS were found with a stress-induced anhedonia model. Here, we additionally studied the effects of DS on several variables which were ameliorated by other insulin receptor sensitizers in mice. Pre-treatment with DS of chronically stressed C57BL6 mice rescued normal contextual fear conditioning, hippocampal gene expression of NMDA receptor subunit NR2A, the NR2A/NR2B ratio and increased REM sleep rebound after acute predation. In 18-month-old C57BL6 mice, a model of elderly depression, DS restored normal sucrose preference and activated the expression of neural plasticity factors in the hippocampus as shown by Illumina microarray. Finally, young naïve DS-treated C57BL6 mice had reduced depressive- and anxiety-like behaviors and, similarly to imipramine-treated mice, preserved hippocampal levels of the phosphorylated (inactive) form of GSK3 beta that was lowered by forced swimming in pharmacologically naïve animals. Thus, DS can ameliorate behavioral and molecular outcomes under a variety of stress- and depression-related conditions. This further highlights neuronal insulin signaling as a new factor of pathogenesis and a potential pharmacotherapy of affective pathologies.

## Introduction

Central insulin receptor signaling is important in brain function/dysfunction including cognitive disorders, stress response, and depression. As a member of a subfamily of receptor tyrosine kinases, the neuronal insulin receptor has been shown to be involved in synaptic plasticity, cell differentiation, myelination, and survival (Chiu et al., 2008; Huang et al., 2010a; Lin et al., 2010) and metabolic processes (Govind et al., 2001; Zhao and Alkon, 2001; Freude et al., 2008). Insulin signaling has been found to regulate dopamine-mediated neurotransmission (Williams et al., 2007) and extracellular levels of norepinephrine and serotonin (Daws et al., 2009). The robust density of the neuronal insulin receptor in the hippocampus and cerebral cortex (Mufson et al., 1999; Sun et al., 2010) and its high structural homology in the activation loop segment with TrkB suggest its role in stress response (Krishnan et al., 2007; Kikusui et al., 2009; Spencer et al., 2010). Compromised insulin signaling can result in cognitive deficits (van der Heide et al., 2006; Kuhad et al., 2009), neurodegeneration (Pomytkin, 2012) and depressive-like syndrome (Banks et al., 2012; Gold et al., 2013; Pan et al., 2013).

The latest clinical and translational studies have revealed antidepressant-like effects, increased neuronal mitochondrial biogenesis, decreased neuronal damage and anti-inflammatory properties for compounds that potentiate the binding of insulin to its receptor or its immediate molecular consequences via various mechanisms and are therefore called “sensitizers of the neuronal insulin receptor” (Storozhevykh et al., 2007; Igarashi et al., 2008; Storozheva et al., 2008; Eissa Ahmed and Al-Rasheed, 2009; Mittal et al., 2009; Rasgon et al., 2010; Kemp et al., 2011). Such effects were reported for the thiazolidinediones rosiglitazone and pioglitazone (Saubermann et al., 2002; Ali et al., 2006; Zhao et al., 2006; Asghar et al., 2007; Strum et al., 2007; Eissa Ahmed and Al-Rasheed, 2009; Mittal et al., 2009; Rasgon et al., 2010; Kemp et al., 2011). For instance, rosiglitazone, one of the insulin sensitizers of the thiazolidinedione class, induces an antidepressant-like effect in the tail suspension and forced swim tests in mice, reducing immobilization and floating behavior (Eissa Ahmed and Al-Rasheed, 2009). Similar effects were found for pioglitazone, another insulin receptor sensitizer, which were shown to be NMDA receptor-dependent (Salehi-Sadaghiani et al., 2012; Sharma et al., 2012). Rosiglitazone and pioglitazone were reported to be effective in the treatment of major depressive disorder that was refractory to standard antidepressant treatment and accompanied by insulin resistance (Rasgon et al., 2010; Kemp et al., 2011).

The antidepressant-like effects were also reported for a mitochondrial complex II substrate, Dicholine Succinate (DS) (Cline et al., 2012; Costa-Nunes et al., 2012, 2015). DS was found to dose-dependently stimulate insulin-dependent H_2_O_2_ production of the mitochondrial respiratory chain in cerebellar neurons leading to an enhancement of the insulin receptor via insulin-stimulated autophosphorylation of the insulin receptor kinase at tyrosine residues in neurons, which is a key regulatory event of the insulin receptor function. The effect of DS is dependent on the presence of insulin (Storozhevykh et al., 2007; Storozheva et al., 2008; Shomaker et al., 2010; Persiyantseva et al., 2013).

Our previous studies utilizing a mouse depression model where a depressive-like state is induced by chronic stress and defined by a reduction in reward sensitivity, anhedonia, showed the antidepressant- and anti-anxiety effects of DS in CD1 mice (Cline et al., 2012). As for instance, chronic intraperitoneal administration of DS for 7 days, at 25 mg/kg/day before the onset of a 10-day stress, rescued normal sucrose preference, floating and step-down avoidance learning, as well as hippocampal expression of Insulin-like Growth Factor 2 (IGF-2), a member of the insulin gene family with neurotrophic properties (Chen et al., 2011; Bracko et al., 2012; Basta-Kaim et al., 2014). In other experiments, administration of DS for 7 days in mice at similar doses rescued aging-related decreases of brain N-acetylaspartate/creatine, a marker of neuronal function and viability and the acquisition of hippocampus-dependent tasks in rat models of chronic cerebral hypoperfusion and beta-amyloid peptide-(25–35)-induced toxicity (Storozheva et al., 2008).

Meanwhile, the antidepressant effects of DS were not assessed in other than chronic stress depression model, e.g., in models that mimic a state of learned helplessness which is distinct from hedonic deficit and an important feature of depression (Porsolt and Papp, 1998). Moreover, the possibility might exist that the antidepressant effects of DS could be limited by the conditions induced by stress and will not preclude other origins/manifestations of a depressive-like syndrome. However, the above mentioned efficacy of other insulin receptor sensitizers with regard to measures of helpless behavior, e.g., in the tail suspension and forced swim test, and the ameliorative effects of DS in aged rodents suggest the efficacy of DS in a variety of experimental conditions. Based on this, the current study's objectives were to examine the effects of DS on several behavioral, molecular and EEG variables that were previously characterized as biological correlates of depressive state and adaptive response to stress in mice.

In the first experiment, using a model of stress-induced anhedonia (Strekalova and Steinbusch, 2010; Costa-Nunes et al., 2014; Cline et al., 2015) we investigated whether a pre-treatment in C57BL6J mice with DS, at the dose of 25 mg/kg/day intraperitoneally for 7 days, would improve normal sleep rebound (augmentation) following acute stress, a sign of adaptive stress response (Marinesco et al., 1999; Suchecki et al., 2012; Albu et al., 2014; Keshavarzy et al., 2014), as well as contextual fear conditioning learning that is regarded to be related to the adaptive sleep function (Rolls et al., 2013; Barnes and Wilson, 2014). Also, we studied hippocampal gene expression of NMDA receptor subunit NR2A and the ratio of NR2A/NR2B, whose increases were previously demonstrated to accompany a development of stress-induced anhedonia in the here applied chronic stress model (Costa-Nunes et al., 2014). Notably, changes in the NMDA-receptor mediated transmission were shown to underlie the antidepressant effects of the neuronal insulin sensitizer pioglitazone (Salehi-Sadaghiani et al., 2012).

Next, we have examined the potential antidepressant-like effects of DS in a recently validated model of elderly depression, where naïve 18-month-old C57BL6 exhibit hedonic deficit in a sucrose test, which is reversible by drugs with antidepressant and neuroprotective activity (Malatynska et al., 2012). The effects of 7-day intraperitoneal injections of DS at the dose of 25 mg/kg/day to aged mice were assessed in the sucrose test and Illumina assay of gene expression profiling of the hippocampus and prefrontal cortex.

Finally, we applied a 2-week administration of DS via drinking water at two doses of 25 and 75 mg/kg/day, in young naïve C57BL6J mice, and tested them in 2-day forced swim test. The latter treatment group of mice was also investigated in the novel cage and elevated O-maze, to assess potential changes in their depressive- and anxiety-like behaviors, as well as locomotion / exploration. A 2-week dosing with imipramine via drinking water (2.5 and 15 mg/kg/day) was used as a reference antidepressant treatment in the forced swim test. Additionally, hippocampal levels of the phosphorylated (inactive) form of Glycogen synthase kinase-3beta (pGSK3 beta), a previously determined marker of depressive-like behavior and antidepressant activity in the forced swim test (Markova et al., 2013a, 2014), were evaluated after the exposure of mice to forced swimming and treatment with DS at the dose of 75 mg/kg/day or imipramine at the dose of 15 mg/kg/day.

## Materials and methods

### Animals

Studies were performed using 3.5-month-old male C57BL/6J mice. 3.5-month-old male CD1 mice were used for resident-intruder for social defeat paradigms and 2-5-month-old Wistar rats were used for predator stress. All animals were from the Gulbenkian Institute of Science, Oeiras, Portugal. C57BL/6J mice were housed individually for 14 days before the start of experiments; CD1 mice and rats were housed in groups of five before the experiment and then individually. All animals were under a reversed 12-h light–dark cycle (lights on: 21:00 h) starting from the day of animals' transportation in the laboratory, with food and water *ad libitum*, under controllable laboratory conditions (22 ± 1°C, 55% humidity).

All studies were carried out in accordance with the European Communities Council Directive for the care and use of laboratory animals. A license BH-2007 had been issued by the Ethics Committee on Animal Experimentation of Claude Bernard University of Lyon, in compliance with the decree No.: 03-505-2008 of the French Agriculture Ministry; permission 0421/000/000/2013 was issued by General Directory of Ethical Committee of the New University of Lisbon, in accordance with Portuguese Law-Decrees DL129/92 (July 6th), DL197/96 (October 16th) and Ordinance Port.131/97 (November 7th). This study had been also approved by the ethics committee of Maastricht University for animal research: CPV, DEC-UM 2009-109.

### Study design with chronic stress depression model and EEG analysis of sleep

**Figure 1:**
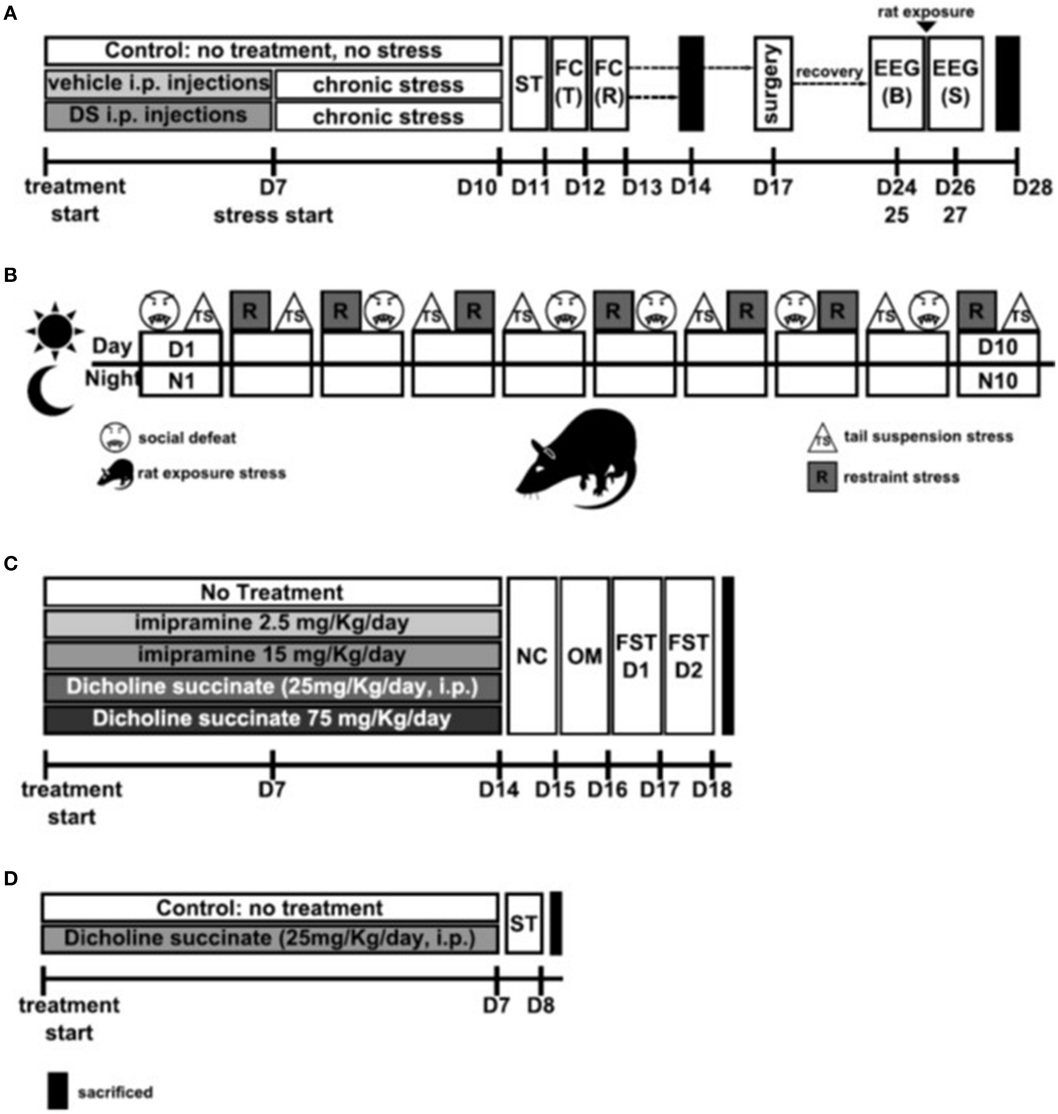
**Study flows. (A) Study of the effects of Dicholine Succinate in a chronic stress model**. Abbreviations: DS, dicholine succinate-treated; ST, sucrose test; FC, fear conditioning; T, training; R, recall test; B, baseline; S, stress; D, day of experiment. **(B)** Schematic of the 10 day chronic stress procedure. Abbreviatons: TS, tail suspension stress; R, restraint stress; D, day; N, night Rat: rat exposure stress. **(C)** Study of the effects of Dicholine Succinate in a model of elderly depression; D: day of experiment. Abbreviations: ST, sucrose test. **(D)** Study of the effects of Dicholine Succinate in naïve mice. Abbreviations: NC, novel cage test; OM, O-maze test; FST D1, forced swim test day 1; FST D2, forced swim test day 2; D, day of experiment.

**Figure 2:**
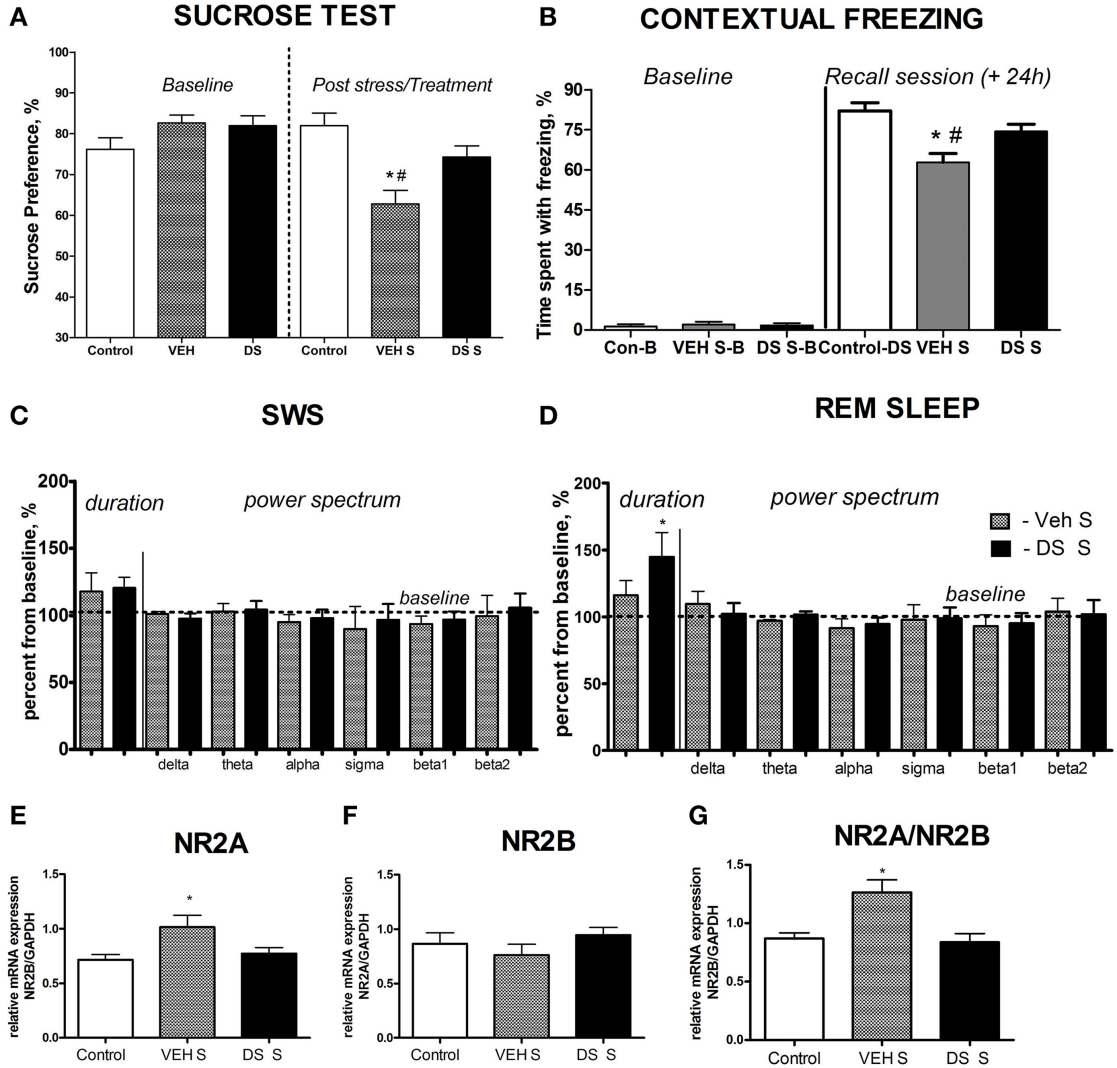
**Effects of Dicholine Succinate in a chronic stress model. (A)** Sucrose preference was significantly lowered in stressed vehicle treated animals as compared to their controls as well as to DS-treated stressed mice (*p* < 0.001 vs. control and DS respectively). **(B)** In contextual fear conditioning, the percentage of time spent with freezing during a baseline (pre-training) period was similarly low in all experimental groups (*p* > 0.05). The vehicle stressed group showed memory loss as indicated by significantly lesser freezing compared to both non-stressed controls (*p* < 0.01) and DS treated stressed mice during a recall session that was evaluated 24 h after a baseline measurement and a training session (+24 h) *p* < 0.01. **(C)** Slow wave sleep was not different between the not treated stressed or DS treated stressed groups (*p* > 0.05). Power spectra analysis revealed no differences between the groups (*p* > 0.05). **(D)** REM sleep was significantly increased in stressed DS treated mice as compared to stressed not treated animals (*p* < 0.05); number of animals per group as indicated above. Power spectra analysis revealed no differences between the groups (*p* > 0.05). **(E)** mRNA expression of NR2A, as well as the NR2A/NR2B ratio **(G)** were elevated in the non-treated stress group, as compared to controls (*p* < 0.05, NR2A; and *p* < 0.01 NR2A/NR2B ratio), but not in stressed DS-treated mice (*p* > 0.05). **(F)** No changes were observed for NR2B mRNA expression. Abbreviations: Con, control group; VEH, vehicle-treated; DS, dicholine succinate-treated; B, baseline conditions; S, stressed. ^*^p < 0.05 vs. controls, #*p* < 0.05 vs. DS-treated group.

Chronic Stress Procedure and Behavioral Testing: Chronic stress and behavioral tests associated with its analysis were performed as described previously (Strekalova et al., 2011, 2013, 2015; Couch et al., 2013). Mice assigned to the stress group were injected with DS (*n* = 20; 25 mg/kg/day, i.p.) or vehicle (*n* = 19; see below) during 1 week prior the stress procedure as described elsewhere (Cline et al., 2012). Control mice (*n* = 7) were not treated. Animals were assigned to three experimental conditions and had similar body weight and baseline sucrose preference (**Figure 2A**). Briefly, animals were exposed to 10 days of chronic unpredictable stress followed by behavioral testing using a two-bottle sucrose preference test (performed on Day 11; see below), as well as a contextual fear conditioning learning task (performed on Days 12 and 13) that was carried out as described previously (Strekalova et al., 2003; Vignisse et al., 2014; see below). Twenty four hours after the termination of behavioral testing, on Day 14, a fraction of stressed vehicle- (*n* = 8), DS-treated (*n* = 6) and control (*n* = 7) mice, were sacrificed for brain dissection and subsequent RT PCR analysis of NMDA receptor subunits NR2A and NR2B. Another fraction of stressed vehicle- (*n* = 7), DS-treated (*n* = 6) mice were subjected to a sleep rebound paradigm and EEG recording (see below, Figure 1A). Remaining mice were used for other assays not reported in the current work. In addition, non-stressed mice that either received DS or not (*n* = 15 in each group) were studied in a sucrose preference test before and after 10 days following the dosing with DS was performed as described above.

The chronic stress procedure employed in this study comprised night time rat exposure and day time application of two of three stressors: a social defeat, restraint stress and tail suspension, a combination of which was applied in a semi-random manner (Figure 1B; Strekalova and Steinbusch, 2010; Couch et al., 2013). Briefly, between the hours of 09:00 and 18:00 two stressors per day were employed in the following sequence: social defeat for 30 min, restraint stress for 2 h and tail suspension for 40 min with an inter-session interval of at least 4 h. This procedure induces anhedonia in a considerably shorter time than previously validated models by increasing the daytime stress load. Details of rat exposure and chronic stressors can be found in supplementary materials.

Sleep rebound paradigm and EEG recording: One week after the termination of stress procedure, another fraction of vehicle-injected control and chronically stressed animals received surgically implanted electrodes. Animals were anaesthetized using a ketamine-xylazine mixture (respectively, 4 and 75 mg/kg, i.p.), placed in a stereotaxic frame and body temperature was maintained at 36.5–37°C by use of a homoeothermic blanket. Two electrodes (length, 2 mm; diameter, 500 μm; both stainless steel and connected to Teflon-insulated wires) were placed into the left and right frontal cortices (2 mm lateral and anterior to Bregma (Cespuglio et al., 1999). Two additive electrodes were placed into the left and right parietal cortices (2 mm lateral to the midline at the midpoint between Bregma and lambda (Cespuglio et al., 1999) for electroencephalographic recordings (EEGs). To obtain electromyograms (EMGs), three electrodes (active length, 1 mm; diameter, 500 μm, all stainless steel and connected to Teflon-insulated iron wires) were inserted between two neck muscle layers. After placement, all electrodes were soldered to two miniature five-pin connectors (Sei 3D, Lyon, France) and the entire assembly anchored to the skull using Super-Bond glue (Sun Medical, Co., Shiga, Japan) and dental acrylic resin (Ivoclar, Lyon, France). Together, four electrodes were implanted within the frontal and parietal cortex, and one electrode in the neck muscle.

After 1 week of recovery that was combined with an acclimatization procedure to the EEG recording chambers, where mice were connected to recording cables and placed individually in plastic cages in a sound-insulated room (ambient temperature, 22 ± 1°C; light-dark cycle 12 h–12 h, water and food *ad libitum*). Thereafter, starting at 16.00, 48-h EEG polysomnographic recordings (Embla, Medcare, Iceland) were performed in these mice during baseline conditions and immediately following the 6-h rat exposure stress (from 10.00 to 16.00) as previously described (Couch et al., 2015).

For acute predation stress, mice were introduced into specialized cylindrical containers allowing visual and odor contact (Costa-Nunes et al., 2014), which were placed into the rat home cage. Visual scoring of digitized EEG and EMG traces (EEG filtering 0.5–49.9 Hz and EMG filtering 15–49.9 Hz) was performed over 10 s bins to quantify the number and duration of sleep-wake episodes and the circadian scheduling of sleep-wake states as described elsewhere (Cespuglio et al., 2012; Strekalova et al., 2015). EEG power spectra (Somnologica software, Medcare, Iceland) were also characterized. To this end, EEG traces sampled at 100 Hz were subjected to fast-Fourier transformation (256 points, computational window 2.56 s, and 50% overlap). Spectra were averaged over 10 s bins and divided into five adjacent bands: delta, 0.5–4 Hz; theta, 4–8 Hz, alpha, 8–11.5 Hz, sigma, 11.5–14.5 Hz; beta-1, 14.5–18.6 Hz, and beta-2, 18.6–30 Hz, and expressed as percentages of total band power (0–49.9 Hz).

The duration of slow wave sleep (SWS) and Rapid Eye Movement (REM) sleep was averaged for 48-h baseline and 48-h after-challenge periods for each animal. Because of well-known inter-individual variability in sleep parameters of rodents, to evaluate the effects of a predation stress, the EEG data for that period were expressed in percent from the averaged baseline for each mouse, as described earlier (Cespuglio et al., 2012; Strekalova et al., 2015).

### Study design with model of elderly depression

In this experiment, we examined the potential effects of DS on the consumption of palatable 1%-sucrose solution by old mice using a two-bottle sucrose preference test. A decrease in sucrose intake and preference over water is generally taken as a putative sign of anhedonia in rodents (Harro et al., 2001; Willner, 2005) and was shown to be decreased in 18-month-old C57BL6 mice; imipramine and the neuroprotective drug dimebon were shown to reverse this deficit (Malatynska et al., 2012). It was investigated whether DS administered to 18-month-old mice (*n* = 9) for 1 week at the dose 25 mg/kg/day would affect the parameters of the sucrose preference test, in comparison with a group of mice of the same age that did not receive such a treatment (*n* = 8). The dose of DS was based on previous studies with CD1 mice, in which its administration, with the above-indicated dosing scheme, effectively reduced the stress-induced decrease in sucrose intake and preference, floating behavior and alteration of hippocampal gene expression typical of the subgroup of mice susceptible to anhedonia (Cline et al., 2012). Twenty four hours after the termination of the sucrose test, mice were sacrificed and their hippocampal formation and prefrontal cortex were isolated for subsequent gene expression profiling using Illumina assay as described elsewhere (Markova et al., 2013b; see also below and Supplementary Material; Figure 1C).

### Study design with tests for anxiety- and depressive like behavior in naïve mice

Three-months-old male C57Bl6J mice received normal water (control group), imipramine (2.5 or 15 mg/kg/day) or dicholine succinate (DS, 25 or 75 mg/kg/day; Buddha Biopharma Ltd, Helsinki, Finland; both compounds were dissolved in drinking water) for2 weeks (*n* = 15 in each group), and were tested for a depressive-like behavior in a 2-day forced swim test (Malatynska et al., 2012; Markova et al., 2013a, 2014; Costa-Nunes et al., 2015). Prior this testing, mice that received DS at the dose 75 mg/kg/day were additionally compared with control animals in a novel cage test (Strekalova et al., 2004; Strekalova and Steinbusch, 2010) and elevated O-maze (Cline et al., 2012; Malatynska et al., 2012), in order, in particular, to rule out potential confounds in the assessment of floating behavior (*n* = 10 from each group was tested; Figure 1D). Because other studies on mice revealed no effects at the dose of 25 mg/kg/day of DS on the parameters of anxiety and locomotion (Cline et al., 2012), animals from the current experiment treated with this dose were not examined in additional assays.

Since previous studies revealed a decrease of hippocampal pGSK3 beta levels to be a marker of depressive-like behavior in a 2-day forced swim test that was preserved by an antidepressant treatment including imipramine (Markova et al., 2014), we have chosen to study whether this variable is sensitive to the effects of DS treatment as well. Therefore mice, subjected to a 2-day FST test and received DS at the dose 75 mg/kg/day, or imipramine at the dose 15 mg/kg/day or remaining untreated, were sacrificed 10 min after the second swimming session for subsequent isolation of the hippocampus and ELISA assay (see below and Supplementary Material, Figure 1D). An additional group of naïve control animals that were not subjected to FST, was sacrificed and analyzed as well.

### Behavioral tests

#### Sucrose test

In order to assess the hedonic state of mice, they were given a free choice for 8 h (between 9.00–17.00 h) of two drinking bottles; one with 1%-sucrose solution, and another with tap water, as described elsewhere (Strekalova et al., 2011, 2015). To prevent possible effects of side-preference in drinking behavior, the position of the bottles in the cage was switched after 4 h. Special precautions have been made in order to minimize the spillage of liquids and error of measurement during sucrose test. The consumption of water, sucrose solution and total intake of liquids was estimated simultaneously in the control and experimental groups by weighing the bottles. Percentage preference for sucrose was calculated using the following formula: Sucrose Preference = [Volume of Sucrose solution/(Volume of Sucrose solution + Volume of Water)] × 100%.

#### Fear conditioning test

The test procedure was adapted from a previously described protocol (Strekalova et al., 2003; Vignisse et al., 2014). The apparatus (Evolocus LLC Tarrytown, NY, USA and Technosmart, Rome, Italy) consisted of a transparent plastic cubicle (25 × 25 × 50 cm) with a stainless-steel grid floor (33 rods 2 mm in diameter). A single alternating electric current (AC, 50 Hz; 0.7 mA) was delivered after a 2-min acclimatization period. Freezing behavior was scored by visual observation during an acclimatization phase and a test of memory recall that was carried out 24 h later. The freezing episode was defined by a complete lack of movement other than respiration accompanied by the occurrence of a specific posture of tension with the tail in a straight and tense position, as described previously (Fleischmann et al., 2003; Strekalova et al., 2003; Vignisse et al., 2014). The occurrence of freezing behavior was assessed every 10 s for 180 s; each 10-s score was assigned to a freezing or non-freezing period, and the percentage of time spent in freezing was calculated. During delivery of foot shocks, the reaction of the animals was closely observed and rated using a 3-grade score system as maximal (jumping and squeaking), intermediate (jumping only), or modest (running) (Strekalova et al., 2001). After delivery of the current, the mouse was immediately placed back in the home cage.

#### Forced swim test

The Porsolt forced swim test has been used as described elsewhere (Malatynska et al., 2012; Couch et al., 2013). Mice were subjected to two 6-min swimming sessions spaced 24 h apart in a transparent cylinder (Ø 17 cm) filled with water (+23°C, water height 13 cm, height of cylinder 20 cm, illumination intensity 25 Lux). Floating was defined by the absence of any directed movements of the animals' head and body and was scored manually; criteria of scoring were previously validated using Noldus EthoVision XT 8.5 (Noldus Information Technology, Wageningen, The Netherlands) and CleverSys (CleverSys, Reston, VA, USA). Using this method, the latency of the first episode of floating and the duration of floating behavior were recorded during the 6-min swimming session on the second day of the test. Latency to begin floating was scored as time between introduction of the animal into the pool and the first moment of complete immobility of the entire body for a duration of >3 s. The total time spent floating was scored for the entire duration of the test using post-test video footage.

#### Elevated O-maze

The apparatus (Technosmart, Rome, Italy), which consisted of a circular path (runway width 5.5 cm, diameter 46 cm), was placed 20 cm above the floor. Two opposing arms were protected by walls (height 10 cm), and the illumination strength was 5 Lux. The apparatus was placed on a dark surface in order to reduce reflection and maintain control over lighting conditions during testing. Anxiety-like behavior was assessed using previously validated parameters that were scored manually as described elsewhere (Strekalova and Steinbusch, 2010; Costa-Nunes et al., 2014; Cline et al., 2015). Mice were placed in one of the closed-arm compartments of the apparatus. The latency of the first exit to the anxiety-related open compartments of the maze, the total duration of time spent therein, the number of risk assessment exploratory events and the number of exits to the open arms were scored during a 5-min observation period. The risk assessment exploratory events were defined by the stretching of the head and a body out of the area protected by the walls toward open arm zone, combined with exploratory pose and movements, directed to the edges of the maze. Half of the body and back limbs of a mouse stayed in the close arm area during these events.

#### Novel cage test

The novel cage test was performed to assess vertical activity in a new environment (Strekalova and Steinbusch, 2010; Couch et al., 2013). Mice were introduced into a standard plastic cage (21 × 21 × 15 cm) filled with fresh sawdust. The number of exploratory rears per each minute was counted under red light during a 5-min period.

### Dosing

The current reference antidepressant treatment was selected because of its effects in lowering the rate of stress-induced anhedonia over other methods of delivery and doses of antidepressants (Costa-Nunes et al., 2012, 2014; Strekalova et al., 2013; Cline et al., 2015). Previous experiments revealed an antidepressant-like effect of 1-week pre-treatment with daily i.p. injections of DS (25 mg/kg/day) in CD1 mice for stress-induced depressive-like changes (Cline et al., 2012). Likewise, here DS was administrated during 7 consecutive days to young mice preceding chronic stress or to 18-months-old mice preceding sucrose test, at the above-indicated dose. DS, provided by Buddha Biopharma Ltd (Helsinki, Finland), was dissolved in water for injections. The volume of DS and vehicle injections was 0.01 ml/g body weight 0.01 ml/kg.

In a study of young non-stressed mice exposed to a battery of tests for emotionality, DS was applied via drinking water at the doses of 25 and 75 mg/kg/day. In this study, imipramine (Sigma-Aldrich, St. Louis, MO, US) was administrated via drinking as well. It was dissolved in tap water; the solution was freshly prepared every 2–3 days. Dosage for imipramine was set at 2.5 or 15 mg/kg/day. Since imipramine is light sensitive, bottles were protected by aluminum covers. The calculation of the concentration of DS and imipramine in drinking water was based on the previously evaluated mean volume of daily water consumption in C57BL6J mice that was about 3.0 ml and on the dosage of treatment.

### Brain dissection and quantitative RT-PCR (qPCR)

Mice were killed by cervical dislocation and their brains were dissected. RNA extraction was performed from microdissected snap-frozen hippocampi using RNeasy RNA extraction kit with DNase I treatment, as previously described (Qiagen, Hilden, Germany; Couch et al., 2013; Costa-Nunes et al., 2014). Using random primers and Superscript III transcriptase (Invitrogen, Darmstadt, Germany), 1 μg total RNA was converted into cDNA. The expression levels of NR2A and NR2B as well as the housekeeping gene glyceraldehyde-3-phosphate dehydrogenase (GAPDH), that was used as a reference gene for quantification, were evaluated with TaqMan probes and the CFX96 Real-time System (BioRad, Hercules, CA, USA). Cycling conditions and sequences of primers used are indicated in the Supplementary data.

### Illumina assay

Gene expression profiling was performed using Illumina technology (Northwestern Chicago University, USA) with the hippocampi obtained from 18-months old mice (drug-naïve or treated with DS); five animals per group were analyzed. Total RNA samples were hybridized to IlluminaBeadChips (MouseRef-8 v2 Expression BeadChip; Illumina, Inc. San Diego, CA, USA) which were prepared using the IlluminaTotalPrep RNA Amplification kit (Applied Biosystems/Ambion, Carlsbad, CA, USA); the samples were assigned to the chips in random order with the constraint that no two samples from the same group were assigned to the same chip, to avoid confounding of experimental groups with the chips. Microarray data were analyzed using standard analysis procedures, which included assessment of the overall quality of array data and statistical evaluation of differentially expressed genes. Once the quality of array data was confirmed, the Gene Chip Operating System (Illumina, Inc. San Diego, CA, USA) was used to calculate signal intensities, detection calls, and their associated P values for each transcript on the array. Gene expression was normalized to the expression of the housekeeping gene, beta-actin, due to its stable expression, and calculated as percent mean of the control group of young mice. Differences in gene expression between groups were evaluated using unpaired two-tailed *t*-test.

Illumina data were imported into Partek Genomics Suite and quantile-normalized. Arrays that appeared as outliers on PCA were removed from the dataset. Comparisons between experimental groups were carried out in Partek-GS using ANOVA with appropriate contrasts. *P*-values were adjusted for multiple testing using step-up False Discovery Rate (FDR). The following criteria were used to select differentially expressed genes at different stringency levels: Strict: FDR < 0.05 and |fold change| > 2; Medium: FDR < 0.1 and |fold change| > 1.5; Loose: unadjusted *p*-value < 0.001 and |fold change| > 1.3, Very loose: unadjusted p values < 0.01 and no fold change threshold (only used when more stringent selection criteria yielded zero or very few hits). In the current analysis, “medium” criteria were applied.

### ELISA of pGSK3 beta

Hippocampus was homogenized in buffer containing 10 mM Tris (pH7,4), 100 mM NaCl, 1 mM EDTA, 1 mM EGTA, 1 mM NaF, 20 mM Na_4_P_2_O_7_, 10% glycerol, 2 mM Na3VO4 in the presence of a protease inhibitor cocktail (Sigma, USA). The GSK-3β [pS9] ELISA kit (Invitrogen Corporation, USA) was used for detection and to quantify the level of GSK-3beta protein phosphorylated at serine residue 9. After three incubations according the instruction manual, a signal intensity provided by monoclonal capture antibody specific for GSK-3β that has been coated onto the wells, was evaluated at 450 nm using a plate reader (Wallac 1420 VICTOR, USA). The results were normalized to total protein level in tissues homogenates, which was determined by the biuret assay; bovine serum albumin was used as a standard (for further details, see Supplementary data).

### Statistics

Data were analyzed with GraphPad Prism version 5.0 for Windows (San Diego, CA). Unpaired two-tailed test was used to compare two groups; One-Way ANOVA was used followed by Tukey's, or Dunnett's *post-hoc* comparison tests was applied to compare three or more groups. Repeated measurements with non-parametric data were evaluated with Wilcoxon test. The level of confidence was set at 95% (*p* < 0.05) and data are shown as mean ± SEM.

## Results

### Dosing with dicholine succinate preserves normal hedonic status and fear conditioning in a chronic stress paradigm

At the baseline, there was no difference in sucrose preference between the groups (*p* > 0.05, *q* = 0.25, Tukey, Figure 2A). Following a chronic stress paradigm, ANOVA revealed a significant difference for sucrose preference [*F*_(5, 87)_ = 8.608, *p* < 0.0001]. *Post-hoc* analysis showed that only the vehicle-treated stressed group had a significant reduction in sucrose preference compared to controls (*p* < 0.001, *q* = 5.53, Tukey) as well as to their DS-treated stressed counterparts (*p* < 0.05, *q* = 4.55, Tukey, Figure 2A), indicating that treatment with DS was able to preclude a hedonic deficit. Sucrose preference was similar in non-treated non-stressed mice (76.22 ± 2.84%) and DS-treated non-stressed mice [82.01 ± 3.1; *p* = 0.193; *t*_(12)_ = 1.381; unpaired two-tailed *t*-test].

During training in the fear conditioning model, control, vehicle-treated stressed and DS-treated stressed groups had a similar percent of mice expressing responses to foot shock: maximal (45, 50, and 55%, respectively), intermediate (30, 25, and 25%, respectively), and a modest response to the foot-shock (25, 25, and 20%, respectively; *p* > 0.05, exact Fischer test). Baseline rates of freezing behavior measured during training were minimal and did not differ between the three groups either (control vs. vehicle: *p* > 0.05 *q* = 0.25, control vs. DS: *p* > 0.05, *q* = 2.09; data not shown, Tukey); together, suggesting their similar behavior under untrained conditions.

Analysis of freezing behavior during a recall session using ANOVA and Tukey *post-hoc* test revealed a significant difference between the groups [*F*_(5, 68)_ = 4.724, *p* = 0.0009] and showed that the vehicle-treated stressed group had significantly less freezing as compared to their counter parts control (*p* < 0.01, *q* = 5.56, Figure 2B) and DS-treated stressed mice (*p* < 0.01, *q* = 5.27, Figure 2B).

### Effects of dosing with dicholine succinate on sleep parameters of chronically stressed mice in acute-stress sleep rebound paradigm

A fraction of mice exposed to chronic stress, was implanted with electrodes and, after a recovery period, was habituated to the recording chamber and connection to the cables and then subjected to a 48 h EEG registration. In order to assess the effects of acute stress on chronically stressed mice that were either treated with DS, or remained pharmacologically naïve, the recording procedure was interrupted for a 6-h rat exposure stress and then re-started for another 48 h. The duration of SWS and REM sleep was averaged for 48-h baseline and after-predation periods for each animal. Because of well-known inter-individual variability in sleep parameters of rodents, EEG data that were obtained after the predation period, were expressed as percent from the averaged baseline values.

Both groups had an increase of the duration of SWS and REM sleep after acute predation stress in comparison to baseline values (stressed non-treated group: *p* = 0.0158,*W* = 28.0 and *p* = 0.0469, *W* = 24.0, stressed DS-treated group: *p* = 0.0313,*W* = 21.0 and *p* = 0.0255,*W* = 28.0, Wilcoxon). The duration of REM sleep, normalized to baseline, was significantly longer in the DS-treated stressed group compared with the pharmacologically naive stressed group [*t*_(10)_ = 2.478, *p* = 0.0327, unpaired two-tailed *t*-test]; however, no differences were seen for SW sleep [*t*_(11)_ = 0.3451, *p* = 0.7366, unpaired two-tailed *t*-test, Figures 2C,D]. Thus, DS-treated stressed mice demonstrated enhanced REM sleep rebound following acute stress, a sign of adaptive stress response, in comparison with vehicle-treated stressed animals. Power spectra analysis revealed no changes in comparison to baseline measures in both challenged groups (*p* > 0.05, Wilcoxon) and no differences between the groups challenged with a predator stress (*p* > 0.05, unpaired two-tailed *t*-test), during SWS nor during REM sleep stages, as expressed in percent from initial baseline values for these animals (Figures 2C,D; power spectra data for baseline and after-predation conditions can be found in Supplementary Table 2).

### Dosing with dicholine succinate prevents stress-induced increases of mRNA of NMDA receptor subunits in the hippocampus of chronically stressed mice

Since changes in the NMDA-receptor mediated transmission were shown to underlie the antidepressant effects of other neuronal insulin sensitizers, we studied hippocampal gene expression of NMDA receptor subunit NR2A and the ratio of NR2A/NR2B, whose increases were previously demonstrated to accompany a susceptibility to stress-induced anhedonia in the here applied chronic stress model (Costa-Nunes et al., 2014). Twenty four hours after the last behavoral test, i.e., on the 5th day after the termination of chronic stress, in accordance with previously established protocols (Strekalova et al., 2011; Cline et al., 2012), animals were sacrificed for the study of hippocampal gene expression of NMDA receptor subunits. The mRNA levels of NR2A were significantly increased in chronically stressed animals which were not treated with DS following chronic stress [*p* < 0.05, *q* = 379, Tukey, *F*_(2,17)_ = 4.010, *p* = 0.0375, ANOVA, Figure 2E]. There was no overall significant changes in the NR2B mRNA expression levels between the groups following chronic stress [*F*_(2, 21)_ = 0.8881, *p* = 0.4264, ANOVA, Figure 2F]. Untreated stressed mice had an increased ratio of NR2A/NR2B as compared to both controls (*p* < 0.05, *q* = 4.70, Tukey) and the DS stressed groups [*p* < 0.05, *q* = 4.62, Tukey, *F*_(2, 17)_ = 7.625, *p* = 0.0043, ANOVA Figure 2G].

### Effect of dosing with dicholine succinate on sucrose preference of old mice

**Figure 3:**
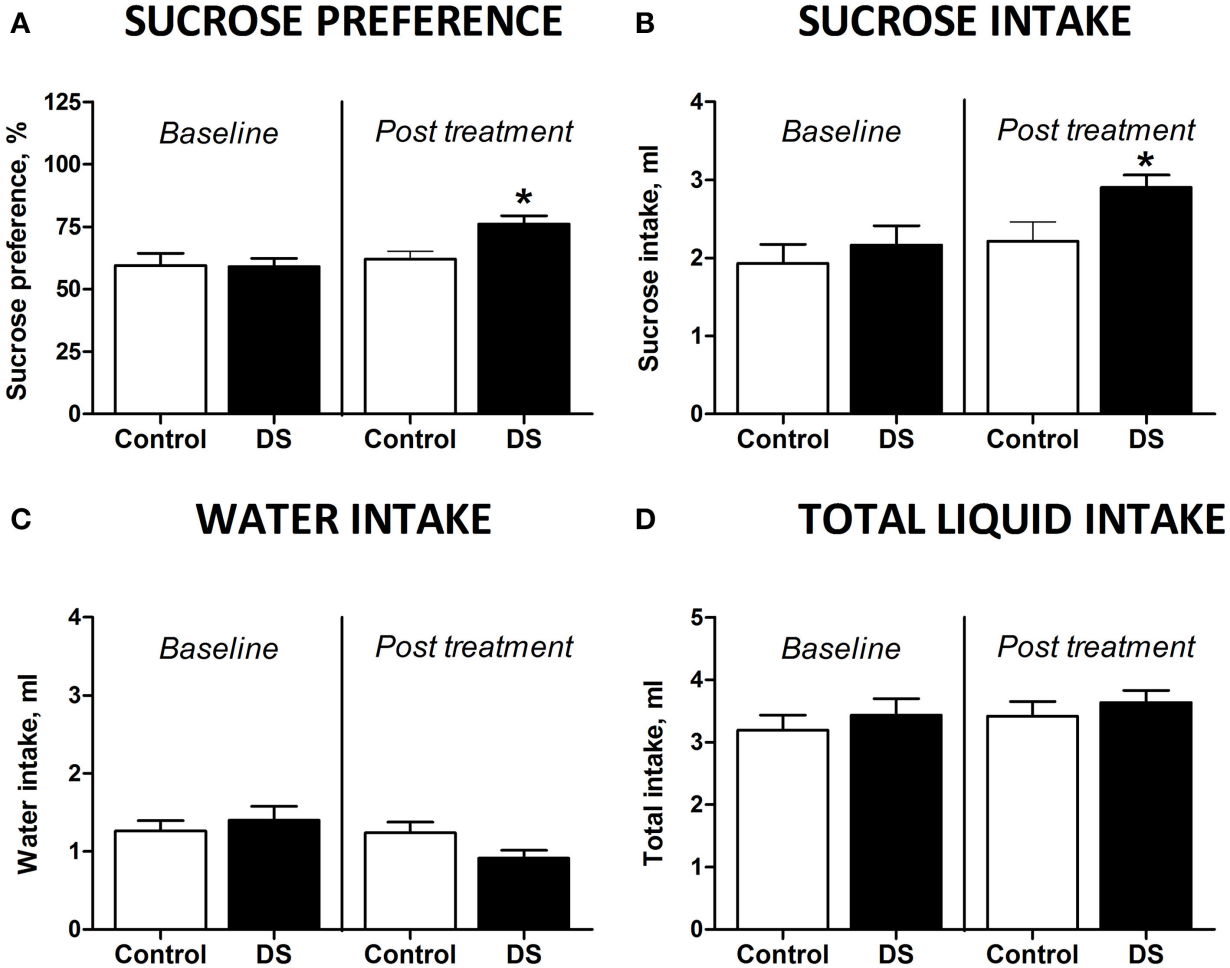
**Effects of Dicholine Succinate in a model of elderly depression. (A)** In 18 month aged animals, only the DS treated group showed an increase in sucrose preference (^*^p < 0.05 vs. controls) while non-treated animals had no increased sucrose preference (*p* > 0.05). **(B)** Total sucrose intake was not changed in animals without treatment (*p* > 0.05) while DS-treated animals had an increase in total sucrose intake (^*^p < 0.05 vs. controls). **(C)** No groups showed any difference for total water intake (*p* > 0.05, not treated and *p* > 0.05, DS). **(D)** Also there were no differences in the total liquid consumption for the not treated animals nor for the DS treatment group (*p* > 0.05).

At the baseline, there was no difference in sucrose preference between the groups [*t*_(7)_ = 0.4509, *p* = 0.6657, unpaired *t*-test]. Animals, which did not receive treatment, aged 18 months showed no differences in preference for sucrose [*t*_(7)_ = 0.4509, *p* = 0.6657, paired two-tail *t*-test Figure 3A] or in sucrose intake [*t*_(7)_ = 0.8845, *p* = 0.4058, paired two-tailed *t*-test, Figure 3B] between two repeated assays of sucrose test. However, sucrose preference and intake of sucrose was significantly increased after dosing with DS [*t*_(7)_ = 2.656, *p* = 0.0327, Figure 3A and *t*_(8)_ = 2.359, *p* = 0.0461, paired two-tailed *t*-test, Figure 3B; respectively]. None of the groups showed any differences for water intake [*t*_(8)_ = 1.099, *p* = 0.3038, control; *t*_(8)_ = 1.850, *p* = 0.1015, DS, paired two-tailed *t*-test, Figure 3C]. Total liquid consumption was also not changed in any of the groups [*t*_(8)_ = 0.8135, *p* = 0.4395, control; *t*_(8)_ = 0.7358, *p* = 0.2414, DS, paired two-tailed *t*-test, Figure 3D].

### Gene expression profiling of the hippocampus and prefrontal cortex of old mice treated with dicholine succinate

**Figure 4:**
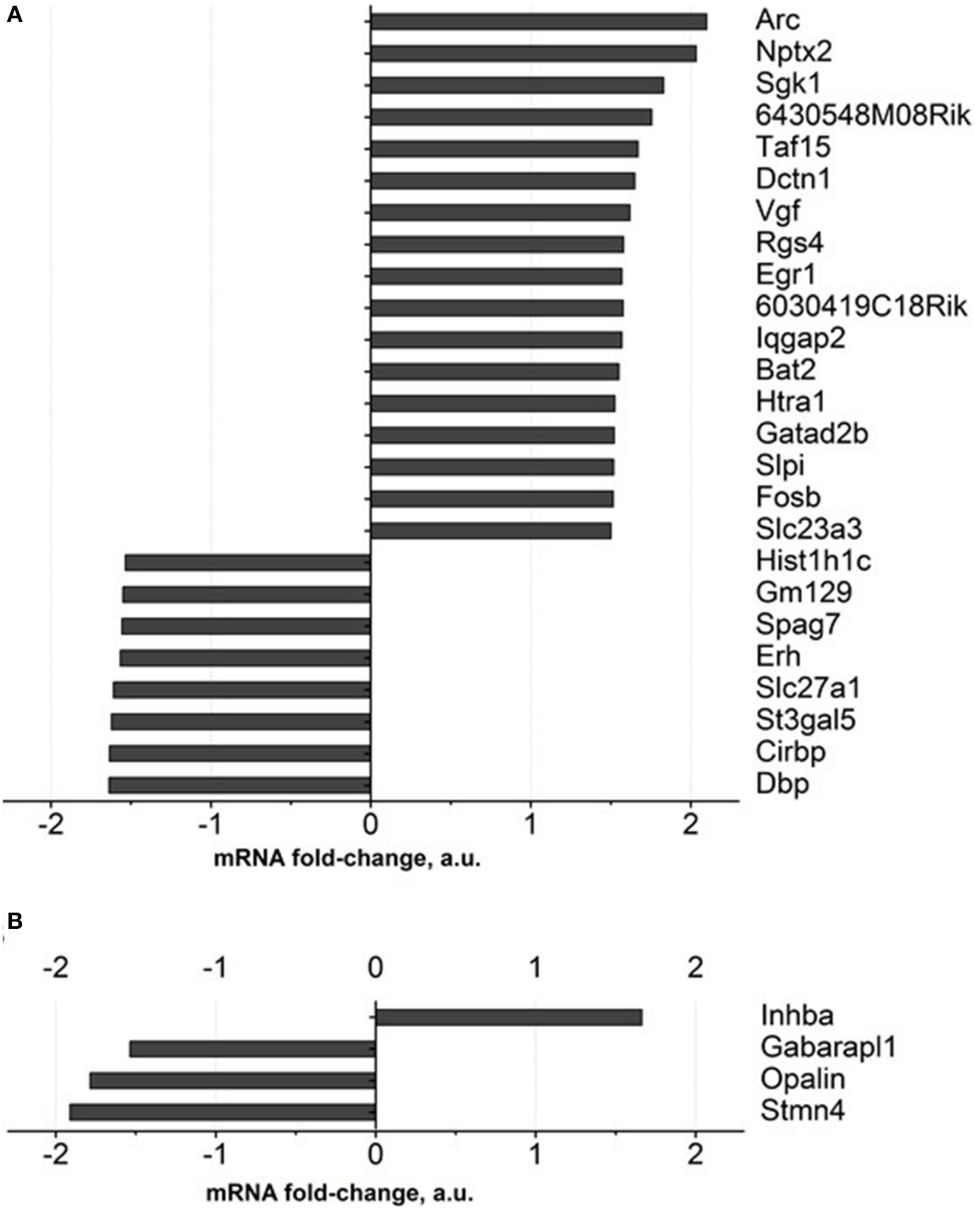
**Gene expression profiling of brain of old mice treated with Dicholine Succinate**. A significant change in over 1.5 folds from vehicle-treated aged control as found with 27 genes in the **(A)**hippocampus and 4 genes in the (**B)** prefrontal cortex. For the criteria of gene selection, see the text.

Gene expression profiling of the hippocampus of DS-treated 18-month-old mice revealed expression changes in 27 genes, in comparison to a control group, for over 1.5 fold, and FDR was <0.1. Among these genes are those involved in neuronal synaptic plasticity: Arc and Nptx2, SGK1, Taf15, Vgf, Egr1, Gatad2b; all of them were up regulated (Figure 4A, Supplementary Table 3). Apart from them, genes encoding ascorbate transporter Slc23a3, regulator of axonal transport Dctn1, serine protease Htra1, serine proteases inhibitor: Slpi were up-regulated as well. The functions of the proteins encoded by 6430548M08Rik and 6030419C18Rik genes were not described in the literature. Functional categories of down-regulated genes in DS-treated old animals constitute genes that regulate sleep and circadian rhythm: Gm129, Cirbp and Dbp, as well as ascorbate transporter Slc23a2, fatty acids transporter Slc27a1.

As for the prefrontal cortex, four genes whose expressions were significantly changed according to the criterion described above were detected. One gene was up regulated (Inhba) while three were down regulated (Figure 4B, Supplementary Table 3).

### Effects of dosing with dicholine succinate of naïve mice: changes in depressive-, anxiety-like behaviors and hippocampal levels of phosphorylated GSK3 beta

**Figure 5:**
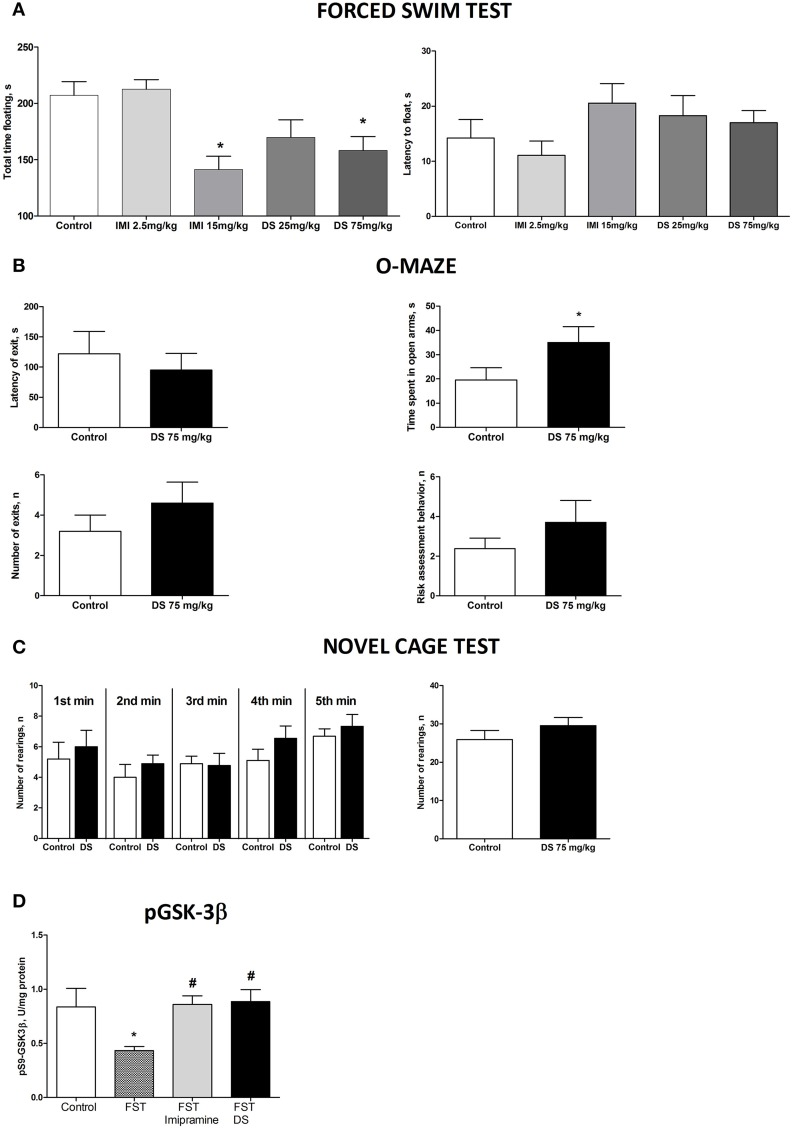
**Study of the effects of Dicholine Succinate in naïve mice. (A)** No differences were observed in the latency to float for any group (*p* > 0.05) while there was a significant effect of total time spent floating (*p* < 0.005). Tukey's revealed that groups receiving higher treatment does had significantly less floating (*p* < 0.01, imipramine and *p* < 0.05, DS). **(B)** In the elevated O-maze test mice treated with a dose of 75 mg/kg/day of dicholine succinate, displayed significantly longer duration in the open arms, with no significant changes to a latency to exit, total number of exits, or risk assessment behavior in comparison with control animals (*p* < 0.05). **(C)** No changes were observed between DS treated animals and controls at any time point (*p* > 0.05, see the text) or for total rearings (*p* > 0.05). **(D)** Tukey's revealed a significant reduction in pGSK-3 beta for untreated animals subjected to FST compared to naive control (*p* < 0.05). There was no difference for imipramine-treated mice subjected to FST (*p* > 0.05) or DS-treated mice subjected to FST (*p* > 0.05), in comparison with naive control group. pGSK-3 beta levels were also significantly lower in untreated animals exposed to FST as compared to imipramine treated subjected to FST (*p* < 0.05) and DS treated subjected to FST (*p* < 0.05) groups. Abbreviations: DS, dicholine succinate-treated; FST, subjected to a forced swim test. ^*^p < 0.05 vs. controls, ^#^*p* < 0.05 vs. FST non-treated group.

In the forced swim test (FST), a One-Way ANOVA revealed no changes between the groups in the latency to float and a significant effect over the total time spent floating [*F*_(4,70)_ = 1371, *p* = 0.2528; *F*_(4,70)_ = 6.36, *p* = 0.0002, respectively; Figure 5A]. A *post-hoc* Dunnett's test showed no significant differences between treated animals and a control group for the latency to swim, whereas the duration of immobility was significantly decreased in animals receiving higher doses of imipramine (15 mg/kg/day) and dicholine succinate (75 mg/kg/day) in comparison with controls (*p* < 0.01, *q* = 3.79; *p* < 0.05, *q* = 2–81, respectively; Figure 4A). In the elevated O-maze test mice treated with a dose of 75 mg/kg/day of dicholine succinate, displayed significantly longer duration in the open arms, with no significant changes to a latency to exit, total number of exits, or risk assessment behavior in comparison with control animals [*p* = 0.038, *t*_(18)_ = 1.88; *p* = 0.28, *t*_(18)_ = 0.59; *p* = 0.15, *t*_(18)_ = 1.07; *p* = 0.34, t_(18)_ = 0.99, respectively; unpaired two tailed *t*-test; Figure 5B]. In the novel cage test for locomotion/exploration, animals treated with dicholine succinate exhibited unchanged number of rearings in comparison to a control group [first minute: *p* = 0.61, *t*_(17)_ = 0.52; second minute: *p* = 0.40, *t*_(17)_ = 0.86; third minute: *p* = 0.89, *t*_(17)_ = 0.13; fourth minute: *p* = 0.20, *t*_(17)_ = 1.34; fifth minute: *p* = 0.49, *t*_(17)_ = 0.71; total rearings: *p* = 0.27, t_(17)_ = 1.13; unpaired two-tailed *t*-test; Figure 5C].

A One-Way ANOVA reveals significant group differences in the levels of phosphorylated GSK-3beta in the hippocampus of mice subjected to the forced swim test (*p* = 0.0145; *F* = 4.130). A *post-hoc* Tukey test showed a significant reduction in GSK-3beta in untreated animals as compared with to intact control mice (*p* < 0.05, *q* = 3.85). No such decrease was found in animals tested in the forced swim test that were treated with either imipramine or dicholine succinate (imipramine: *p* > 0.05, *q* = 0.21 and dicholine succinate: *p* > 0.05, *q* = 0.43; Figure 5D). Untreated animals subjected to the forced swim test had significantly lower levels of the hippocampal pGSK-3beta levels in comparison to imipramine- and DS-treated animals (*p* < 0.05, *q* = 3.95 and *p* < 0.05, *q* = 4.03, respectively; Tukey *post-hoc* test; Figure 5D).

## Discussion

### Effects of dicholine succinate in a chronic stress model

In the current work, stress exposure lowered sucrose preference in agreement with other reports (Willner et al., 1987; Harro et al., 2001; Krishnan et al., 2007). Stressed mice treated with DS showed no significant change in sucrose preference measured after the 10th day of stress as compared to control animals (Figure 2A), similarly to the effects of classical antidepressants (Costa-Nunes et al., 2014; Cline et al., 2015; Strekalova et al., 2015). Earlier, we have shown in a model of stress-induced anhedonia that the decrease in sucrose preference is paralleled by a reduction in sucrose intake (Strekalova et al., 2004, 2006). Importantly, administration of DS did not alter sucrose test parameters in non-stressed animals ruling out any possible confounding artifacts for sucrose test measurements which could be related to treatment. Thus, the partial preclusion of the stress-induced reduction for sucrose preference by treatment with DS manifests their antidepressant-like activity in our study that is in line with previous findings obtained in a similar model on CD1 mice (Cline et al., 2012).

Treatment with DS prevented stress-induced memory impairment in the fear conditioning task (Figure 2B). Similar freezing scores at baseline and the responses to foot shock in all experimental groups suggest that the deficits, revealed here in the contextual memory performance in mice subjected to stress and their rescue in the DS-treated stressed animals, are unlikely to be due to any distinct sensitivity to the foot-shock or to basal differences in the emotionality in the groups. While a similarity in these parameters in tested mice is a prerequisite of their unaltered acquisition of fear conditioning, the here employed study design does not exclude the ameliorative effect of DS on either or both learning phase(s), acquisition or / and consolidation of contextual memories. Of interest, the stimulation of neuronal insulin receptor is implicated both in memory acquisition and consolidation (Moosavi et al., 2007) suggesting that DS can be involved in two of these processes.

The effects of DS in the mouse fear conditioning paradigm, as previously validated in our model studies of hippocampus-dependent performance in mice (Strekalova et al., 2003; Vignisse et al., 2014), are in line with the ameliorative effects of DS on hippocampus- and cortex-dependent learning in step-though, step-down and Morris water maze paradigms which this drug exerted under pathological conditions of diverse origins (Storozhevykh et al., 2007; Storozheva et al., 2008). Previously reported effects of DS on increased levels of hippocampal IGF-2, brain N-acetylaspartate/creatine, choline acetyltransferase activity (Storozheva et al., 2008; Cline et al., 2012) can attest for the here observed memory preserving effects of DS. Interestingly, choline acetyltransferase activity in the brain was shown to be regulated by neuronal insulin receptor-mediated mechanisms (Hoyer, 2003). Recent evidence for a critical role of IGF2 in inhibitory avoidance learning and adult neurogenesis as shown in the fear conditioning paradigm (Agis-Balboa et al., 2011; Chen et al., 2011; Bracko et al., 2012) can additionally explain the beneficial effects of DS on memory performance in chronically stressed mice. Finally, recently shown activation of insulin receptor-mediated transmission a newly discovered mechanism of augmented neurogenesis (Ziegler et al., 2014), can *per-se* result in pro-neurogenetic and neuroprotective activities that are characteristic for the antidepressants of various classes (Duman and Li, 2012), and, thus, can underlie pro-cognitive and antidepressant effects of DS.

In the present study, we found significantly longer duration of REM but not SWS sleep in chronically stressed DS-treated mice subjected to acute stress of predation (Figure 2C). It is well established that acute stress of various natures, as for instance, immobilization or predation, induces an adaptive effect of sleep rebound, consisting in an increase of the REM stage of sleep and to a lesser extent SWS, this is regarded as one of the important anti-stress mechanisms (Cespuglio et al., 1999; Marinesco et al., 1999; Koehl et al., 2002; Tang et al., 2007; Tiba et al., 2008; Couch et al., 2015). It was shown that stress-induced sleep rebound is decreased during aging (Clement et al., 2003; Descamps and Cespuglio, 2010), development of anhedonia during stress (Couch et al., 2015) and various neurochemical abnormalities associated with neuropsychiatric conditions (Bonnet et al., 2000; Meerlo et al., 2001; Boutrel et al., 2002; Vazquez-Palacios et al., 2004; Albu et al., 2014). While the exact functions of each of the stages of sleep are, as yet unclear, it is claimed that normal REM sleep is a crucial factor of memory consolidation (Rolls et al., 2013; Barnes and Wilson, 2014). Additionally, insulin receptor mediated signaling is regarded as one of the regulatory mechanisms of sleep (Valatx et al., 1999; Kashyap and Defronzo, 2007).

While recent studies suggest that challenging insulin receptor mediated transmission in the brain might have long-term effects lasting for weeks (Hoyer, 2003), we trust that the ameliorative action of DS reported here on sleep rebound is likely to be related to a reduced manifestations of depressive-like changes and stress response in chronically stressed mice that was found to be elevated for weeks in the model applied here when no antidepressant therapies are used (Cline et al., 2015). At the same time, power spectra activity was not changed in DS-treated mice (Figure 2C, Supplementary Table 2) ruling out non-specific general changes in EEG activity of the treatment and suggesting preserved cerebral homeostasis in DS-treated mice that can be compromised by some antidepressants or aging (Cespuglio et al., 1999; Clement et al., 2003).

Our study evidenced preventive effects of DS on stress-induced increases of hippocampal gene expression of NMDA receptor subunits NR2A and the NR2A/NR2B ratio (Figures 2E–G). The increases of these measures were previously shown to accompany elevated anxiety and occurrence of hedonic deficit during stress (Boyce-Rustay and Holmes, 2006; Gao et al., 2010; Calabrese et al., 2012; Costa-Nunes et al., 2014; Pochwat et al., 2014), impulsivity and aggression (Meyer et al., 2004; Bortolato et al., 2012), home cage hyperactivity and a stress-induced elevation in peripheral concentrations of corticosterone (Longordo et al., 2009, 2011; Huang et al., 2010b). A number of findings evidence that hippocampal NR2A and NR2B subunits of the NMDA receptor display fast kinetics in response to CORT and adverse experiences, where changes in gene expression parallel rapid alterations in total and surface protein levels as well as receptor trafficking (Zhang et al., 1997; Tse et al., 2011; Pochwat et al., 2014) suggesting that the changes reported in this study for mRNA levels of the NMDA-receptor subunits reflect its functional alterations.

Other studies demonstrate the importance of NMDA-receptor mediated currents in the antidepressant effects of pioglitazone, as it was discussed above (Salehi-Sadaghiani et al., 2012), which allows speculation that amelioration of depressive-like conditions via enhancement of insulin receptor mediated signaling by different drugs might commonly implicate glutamatergic neurotransmission via this receptor.

### Effects of dicholine succinate in a model of elderly depression

DS-treated 18-month old mice displayed higher sucrose intake and preference than pharmacologically naïve mice of this age, suggesting a normalization of hedonic state by the treatment. Comparable to these changes, similar effects were also demonstrated for treatment with imipramine or the neuroprotective drug dimebon (Malatynska et al., 2012). There was a non-significant reduction of water intake in the DS-treated group that was obviously accounted for compensatory changes in drinking behavior, while total liquid intake was not altered by the treatment (Figures 3A–D). Together, the current findings may be interpreted as a manifestation of an antidepressant-like activity of DS in a model of elderly depression that is in line with previous reports showing that chronic administration of DS counteracts the development of aging-related neurochemical and cognitive deficits in mice (Storozheva et al., 2008) and preserves normal sucrose preference in chronically stressed mice.

Interestingly, Illumina gene expression study showed that among 27 significantly changed genes in accordance to the criterion applied here, 17 were up-regulated: 7 genes from this cohort (41.2%) constituted genes encoding factors of synaptic plasticity (Figure 4A and Supplementary Table 3A). These functions are well established for most of them, such as for immediate early gene Arc, whose activity is regulated by stimulation of insulin receptor in neurons (Kremerskothen et al., 2002; Chen et al., 2014), suppressed by chronic stress (Elizalde et al., 2008, 2010) and increased by antidepressants (Alme et al., 2007; Molteni et al., 2008), immediate-early gene Nptx2 encoding neuronal activity-regulated pentraxin (Narp) that modulates AMPA-receptor functions (O'Brien et al., 1999; Chang et al., 2010), SGK1, which regulates hippocampal postsynaptic density-95 and dendritic growth (Ma et al., 2006; Yang et al., 2006). Also, TAF15 was shown to be implicated in the trafficking of NMDA glutamate receptor (Ibrahim et al., 2013). VGF and Egr1 were found to enhance hippocampal synaptic plasticity and neurogenesis (Thakker-Varia and Alder, 2009). GATAD2B was shown to be required for normal cognitive performance and synapse development (Willemsen et al., 2013).

Another cohort of altered genes in DS-treated mice whose function is well established constitute genes that are involved in the regulation of sleep and circadian activity. These genes include Gm129, a novel regulator of the feedback loop that involves activators and suppressors of circadian regulation (Annayev et al., 2014), Cirbp, a factor of cytokine-regulated expression of clock genes (Lopez et al., 2014) and Dbp, a putative clock-controlled transcription factor, which is increased under sleep deprivation (Wisor et al., 2002).

Remarkably, many of these altered genes are functionally associated with insulin receptor signaling. As for instance, activation of Arc is regulated by insulin receptor in neurons through IRS/Grb2/Raf/Mek/Erk pathway (Kremerskothen et al., 2002); Sgk1 is encoding a kinase that is activated by insulin via PI3-kinase (Lang et al., 2010). Vgf is encoding a neuropeptide, which expression is up regulated by BDNF and insulin (Salton et al., 2000; Busse et al., 2012); Rsg4 plays a role as a negative regulator of insulin-stimulated GLUT4 translocation in adipocytes (Kanzaki et al., 2000). Finally, Htra1 is encoding a protease that regulates the availability of insulin-like growth factors (IGFs) by cleaving IGF-binding proteins (Zumbrunn and Trueb, 1996); FosB is encoding a transcription factor, which its periphery expression is up-regulated by insulin (Coletta et al., 2008).

Notably, DS evoked limited changes in gene expression in the prefrontal cortex. Among four genes, whose expression was significantly changed in this study is at least one factor that was shown to be implicated in the morphological plasticity of the brain and antidepressant response. Inhba encodes a beta A subunit that is shared by glycoprotein families Activins and Inhibins, that were shown to have opposite functions concerning antidepressant mechanisms (Ganea et al., 2012). Activin A, the homodimer of beta A, was shown to exert and acute antidepressant-like effect and increase the formation of synaptic contacts by modulating the dynamics of actin in the neuronal spines (Shoji-Kasai et al., 2007; Ganea et al., 2012). Expression of other genes belonging to various classes of regulators whose functions in the CNS are not well defined are associated with autophagy (Gabarapl1), other structural functions (Opalin) and myelin organization (Stmn4) were also significantly changed. Other significantly altered genes encode molecules whose functions in the brain are not well defined and mostly associated with structural functions and myelin organization (Figure 4B and Supplementary Table 3B).

While gene expression profiling data in this study need verification using additional methods, it is remarkable that many changes are associated with activation of brain plasticity factors and changes in sleep/circadian regulation that are known to be implicated in the pathogenesis of depression and antidepressant treatment (Mellman et al., 2002; Wainwright and Galea, 2013). Moreover, a number of highlighted gene changes were also shown to affect the elements of insulin receptor-mediated signaling that could be expected with the use of compounds that like DS stimulate this processes.

### Effects of dicholine succinate on behavior and hippocampal pGSK3 beta in naïve mice

While the effects of either treatment on the latency to the first episode of floating were not significant, mice treated with higher doses of imipramine and DS had significantly shorter duration of this behavior (Figure 5A). This is in line with recent findings treated with DS via food and tested in the tail suspension and FST and together suggests that this treatment diminishes the symptoms of learned helplessness (Costa-Nunes et al., 2015). Coinciding with these results, another insulin sensitizer, rosiglitazone, was reported to reduce immobilization and floating behaviors in mouse tail suspension and forced swim tests respectively (Eissa Ahmed and Al-Rasheed, 2009). Such effects well documented for other antidepressants and are regarded as a manifestation of antidepressant-like activity (Porsolt and Papp, 1998; Willner, 2005).

Treatment with DS decreased anxiety scores as shown by increased time spent in the open arms of the elevated O-maze indicating its anxiolytic and anti-stress effect (Figure 5B). Such effects are well documented for compounds with anxiolytic activity (Willner, 2005). Elevated anxiety was found to parallel induction of a depressive-like syndrome (Willner et al., 1987; Willner, 2005; Krishnan et al., 2007; Strekalova et al., 2011). No changes in vertical activity were found in the DS-treated group at no time period of the observation in the novel cage test, suggesting a lack of general effects on locomotion and a specificity of the above-described effects on measures of depression- and anxiety-like behaviors (Figure 5C).

Because on one hand, insulin, via the IRS/PI3K/Akt pathway, regulates GSK3-beta activity (Cross et al., 1995) and on another hand, mice exposed to a two-day forced swimming revealed decreased hippocampal levels of phosphorylated at Ser9 (inactive) form of GSK3beta (Markova et al., 2014), we assessed potential effects of DS on the latter measure as well. We found that DS at the dose in which it exerted an antidepressant effect in this test, precluded stress-induced reduction of pGSK3 beta in this study that was comparable to the effect of imipramine. The present finding is in line with an inhibitory effect of insulin on GSK3-beta activity. Given accumulating evidence for similar effects of other antidepressant interventions and, in general, for the role of elevated function of GSK3-beta in promoting mood disorders and neurodegeneration (Doble and Woodgett, 2003; Li and Jope, 2010), these results suggest that the above-described effects on pGSK3beta can underlie an antidepressant and pro-cognitive action of DS. As GSK3-beta plays a key role in the induction of NMDA-receptor-dependent LTD (Peineau et al., 2009; Bradley et al., 2012) the effects of DS on hippocampal gene expression of NR2A subunit of this receptor can be related to the changes in the pGSK3 levels in this study.

## Conclusions

Although a link between DS treatment and its mechanism of action in the distinct mouse models applied here remains to be determined, the present study argues for the potential of DS to generate an antidepressant-like effect in various conditions, including those in which the mechanisms of action of other sensitizers of the insulin receptor are effective. A lack of signs of toxicity of choline succinate in mammals (Shivapurkar et al., 1986; Maekawa et al., 1990) at the dosage ranges of DS found effective in our study favors its potential practical use.

We conclude that the insulin receptor sensitizer DS ameliorates depressive-like features in mice whose induction was associated with chronic stress as well those which were not. In a model of stress-induced anhedonia, DS preserved normal contextual fear conditioning, hippocampal gene expression of NMDA receptor subunit NR2A, the NR2A/NR2B ratio and increased REM sleep rebound after acute predation. In a model of elderly depression, DS restored normal sucrose preference and altered gene expression of 27 genes of the hippocampus and the prefrontal cortex most of which are involved in brain plasticity and sleep/circadian regulation. Finally, young DS-treated C57BL6 mice had reduced signs of learned helplessness through lowered scores of floating, similarly to the imipramine-treated group. Also, like imipramine-treated mice, DS-treated mice demonstrated preserved hippocampal levels of the phosphorylated (inactive) form of GSK3 beta that was lowered by forced swimming in pharmacologically naïve animals. Thus, even though a variety of experimental techniques and determined physiological, behavioral and molecular read-outs of a depressive-like state dose not quite allow a connection these findings to each other, they all point toward an antidepressant-like role for DS at different levels and in different contexts. Consequently, this further highlights the enhancement of insulin receptor signaling as a potential target of pharmacotherapy of depressive disorder, while exactly how this mechanism results due to the effects of DS reported here, remains to be discovered.

## Author contributions

BC and JCN carried out the chronic stress experiment, tissue collection, statistical analysis, prepared the figures and took part in drafting of the manuscript; RC and AS organized and carried out EEG study on chronically stressed mice; YB performed gene expression Illumina analysis; NM and AK performed study in old mice and participated in the ELISA and RT PCR assays; HWMS participated in the design of the study and coordination; KPL participated in the coordination of the study and contributed to the drafting of the manuscript; TS conceived of the study, participated in its design and coordination and drafted the manuscript. All authors read and approved the final manuscript.

### Conflict of interest statement

The authors declare that the research was conducted in the absence of any commercial or financial relationships that could be construed as a potential conflict of interest.
