# Supplementary material for: Dicholine succinate, the neuronal insulin sensitizer, normalizes behavior, REM sleep, hippocampal pGSK3 beta and mRNAs of NMDA receptor subunits in mouse models of depression
Source: Front Behav Neurosci. 2015 Feb 26;9:37. doi: 10.3389/fnbeh.2015.00037 (PMC4341562; doi:10.3389/fnbeh.2015.00037)
Supplement: Supplementary file 1 [file DataSheet1.DOCX]

**Supplementary data**

## *Rat exposure while in a small container*

Mice were introduced into cylindrical containers, which were placed into a rat home cage for 15h (over-night, from 18h00 to 9h00). Containers were made from customized transparent plastic, size 15cm x Ø 8 cm, with holes in covers (Ø < 0.5cm), which ensured protection of the mouse from the rat, but allowed visual and odor contact.

## *Social defeat stress*

Social defeat took place during the dark phase under red light to enable visual control over the resident-intruder confrontation. In a preliminary test, aggressive individuals of the CD1 mouse strain that were able to attack the counter-partners in less than 60 sec without injuring them were selected for this procedure; these animals were introduced in the home cages of mice from the stress group during social defeat sessions for 5 min. During social defeat stress, test mice typically showed flight response, submissive posture and vocalization. Pairs of animals were carefully observed in order to exclude any physical harm. In rare cases of its incidence, overly aggressive individuals were immediately removed from the cage of resident mice. After a 5-min period of social defeat C57BL/6 mice were introduced into small containers and placed inside the CD1 cage, where they stayed for a 3h-period. Thereafter, a 5-min social defeat procedure was repeated again. In order to randomize the procedure, the same pairs of C57Bl6 and CD1 mice were never put together.

*Tail suspension stress*

Mice were submitted to the tail suspension procedure by hanging them by their tails. The procedure was done during the dark phase of the animals’ light cycle.

*Restraint Stress*

Animals were placed inside a plastic tube (internal diameter 26 mm) for 2 h during the dark phase of the light cycle and kept in a dark experimental room.

*Real-time PCR assay*

RNA extraction was performed as previously described (Costa-Nunes et al., 2014). Cycling conditions and sequences of primers used are indicated in *Table 1*.

*Table 1* Cycling conditions and sequences of primers used in RT PCR assay

| **mRNA target** | **Primer sequence (5' → 3')** | | **Annealing**  **Temperature (C°)** |
| --- | --- | --- | --- |
| **NR2A** | Forward | GCTACACACTCTGCACCAATT | 64 |
|  | Reverse | CACCTGATAGCCTTCCTCAGTGA |  |
|  | TaqManProbe | 6-FAM TGGTCAATGTGACTTGGGATGGCAABHQ-1 |  |
| **NR2B** | Forward | CTGCATGCGGAATACAGTC | 56 |
|  | Reverse | TTGATGTAGCCTGGTTCCT |  |
|  | TaqManProbe | 6-FAM CAGAAGCGCATCATCTCTGAGAATBHQ-1 |  |
| **GAPDH** | Forward | TGCACCACCAACTGCTTAG | 54 |
|  | Reverse | GGATGCAGGGATGATGTTC |  |
|  | TaqManProbe | HEX ATCACGCCACAGCTTTCCAGA BHQ-1 |  |
|  |  |  |  |
|  |  |  |  |
|  |  |  |  |

The qPCR was performed in a 25 μl reaction volume containing a 10хPCR Buffer (2.5μl), 25 mmol MgCl2 (4 μl), 10 mmol dNTPs (2μl), specific forward and reverse primers at 20 pmol/μl concentration (1 μl), 5pmol/μl Taq Man probe (1.5 μl), cDNA (2μl), 5 u/μl Taq DNA polymerase (1 μl) (Beagle, st. Petersburg, Russia), and ddH2O (10 μl). All samples were run in duplicate. Cycling was performed at 95 C° for 5 min followed by a 45-cycle amplification at 95°C for 10s, then at the annealing temperature (see Table 1) for 15 s and at the temperature 72°C for 20 s.

Results of the qPCR measurements were expressed as Ct values, where Ct is defined as the threshold cycle of PCR at which amplified product was 0.05 % of normalized maximal signal. We used the comparative Ct method and computed the difference between the expression of the gene of interest and GAPDH expression in each cDNA sample (2−ΔΔ Ct method). Results are expressed as folds of expression compared to the mean values of expression in non-stressed control animals (Couch et al., adapted from Livak and Schmittgen 2001).

*ELISA of pGSK3 beta*

Hippocampus was homogenized in buffer containing 10 mM Tris (pH7,4), 100 mM NaCl, 1 mM EDTA, 1 mM EGTA, 1 mM NaF, 20 mM Na4P2O7, 10% glycerol, 2mM Na3VO4. Protease inhibitor cocktail (Sigma, USA) was added immediately prior to homogenization. The GSK-3β [pS9] ELISA kit (Invitrogen Corporation, USA) was used for detection and quantification of the level of GSK-3β protein phosphorylated at serine residue 9. A monoclonal capture antibody specific for GSK-3β has been coated onto the wells of the 96-well plate provided. During the first incubation, standards of known GSK-3β [pS9] content and unknown samples are pipetted into the wells and the GSK-3β antigen binds to the immobilized (capture) antibody. After washing, a rabbit antibody specific for GSK-3β [pS9] is added to the wells. During the second incubation, this antibody serves as a detection antibody by binding to the immobilized GSK-3β protein captured during the first incubation. After washing, a horseradish peroxidase–labelled anti–rabbit IgG is added. This binds to the detection antibody to complete the four–member sandwich. After a third incubation and washing to remove all the unbound enzyme, a substrate solution (TMB) is added, which is acted upon by the bound enzyme to produce color. The intensity of this colored product is directly proportional to the concentration of GSK-3β [pS9] present in the original specimen. All procedures were done according the instruction manual and the optical density of experimental plates were measured at 450 nm using a plate reader (Wallac 1420 VICTOR, USA). The results were normalized to total protein level in tissues homogenates. Protein concentrations were determined by the biuret assay using bovine serum albumin as a standard (Gornall, A. G., Bardawill, C. J., and David, M. M. (1949) J Biol Chem 177, 751-766).

*Table 2.* EEG power spectra parameters in chronically stressed mice were not affected by DS.

| **Treatment** | **Delta** | **Theta** | **Alpha** | **Sigma** | **Beta 1** | **Beta 2** |
| --- | --- | --- | --- | --- | --- | --- |
| SLOW WAVE SLEEP | | | |  |  |  |
| Vehicle baseline | 39.95±0.5 | 32.15±0.8 | 14.10±0.9 | 3.55±0.4 | 5.04±0.2 | 5.21±0.7 |
| Vehicle stress | 40.40±0.7 | 33.10±1.9 | 13.40±0.8 | 3.19±0.6 | 4.72±0.3 | 5.19±0.8 |
| DS baseline | 41.10±0.8 | 32.60±0.9 | 14.25±1.0 | 3.39±0.3 | 4.88±0.1 | 3.78±0.5 |
| DS stress | 40.01±1.6 | 34.00±2.1 | 13.97±0.9 | 3.28±0.4 | 4.73±0.3 | 4.00±0.7 |
| REM SLEEP | | |  |  |  |  |
| Vehicle baseline | 24.32 ±1.4 | 41.79±0.9 | 17.05±0.7 | 3.56±0.3 | 5.98±0.6 | 7.30±0.7 |
| Vehicle stress | 26.67±2.3 | 40.52±0.45 | 15.60±1.2 | 3.48±0.4 | 5.86±0.5 | 7.87±1.1 |
| DS baseline | 23.65±1.7 | 39.86±1.2 | 17.87±0.9 | 3.64±0.2 | 6.53±0.4 | 8.45±1.1 |
| DS stress | 24.18±1.9 | 40.50±1.0 | 16.90±0.9 | 3.60±0.3 | 6.21±0.5 | 8.61±1.4 |

*Table 3.* **(A)** List of differentially expressed genes in the hippocampus of old mice treated with DS.

*Ratio=(mRNA abundance DISU/ mRNA abundance Control) Unadjusted p value <0.001 and |fold change| >1.5 (see the text)

| **Gene name** | **Gene Symbol** | **Ratio*** | **P-Value** |
| --- | --- | --- | --- |
| Activity-regulated cytoskeleton-associated protein | Arc | 2,097 | 0,0000819 |
| Neuronal pentraxin II | Nptx2 | 2,034 | 0,0000107 |
| Serum/glucocorticoid regulated kinase 1 | Sgk1 | 1,83 | 0,001816 |
| TATA-binding protein-associated factor 2N | Taf15 | 1,67322 | 0,000198648 |
| Dynactin 1 | Dctn1 | 1,65636 | 0,00115384 |
| VGF nerve growth factor inducible | Vgf | 1,62473 | 0,000477935 |
| Regulator of G-protein signaling 4 | Rgs4 | 1,58555 | 0,000175286 |
| Early growth response 1 | Egr1 | 1,57936 | 3,45E-06 |
| IQ motif containing GTPase activating protein 2 | Iqgap2 | 1,57442 | 0,000690179 |
| proline-rich coiled-coil 2A | Bat2 | 1,5569 | 3,56E-05 |
| HtrA serine peptidase 1 | Htra1 | 1,52536 | 1,02E-05 |
| GATA zinc finger domain containing 2B | Gatad2b | 1,52186 | 1,42E-05 |
| Secretory leukocyte peptidase inhibitor | Slpi | 1,51893 | 0,00199586 |
| FBJ murine osteosarcoma viral oncogene homolog B | Fosb | 1,51512 | 6,34E-06 |
| Solute carrier family 23 (ascorbic acid transporter), member 3 | Slc23a3 | 1,50125 | 4,12E-06 |
| Histone cluster 1, H1c | Hist1h1c | -1,53521 | 0,00180782 |
| Circadian Associated Repressor Of Transcription | Gm129 | -1,54957 | 5,65E-05 |
| Sperm associated antigen 7 | Spag7 | -1,55727 | 1,41E-05 |
| Enhancer of rudimentary homolog (Drosophila) | Erh | -1,56672 | 4,47E-07 |
| Solute carrier family 27 (fatty acid transporter), member 1 | Slc27a1 | -1,61057 | 1,63E-05 |
| ST3 beta-galactoside alpha-2,3-sialyltransferase 5 | St3gal5 | -1,62261 | 5,94E-07 |
| Cold inducible RNA binding protein | Cirbp | -1,63479 | 9,62E-06 |
| D site of albumin promoter (albumin D-box) binding protein | Dbp | -1,63823 | 4,01E-10 |
| Oligodendrocytic myelin paranodal and inner loop protein | Opalin | -1,82659 | 0,000166283 |

**(B)** List of differentially expressed genes in the prefrontal cortex of old mice treated with DS.

*Ratio=(mRNA abundance DISU/ mRNA abundance Control) Unadjusted p value <0.001 and |fold change|> 1.5 (see the text)

| **Gene name** | **Gene Symbol** | **Ratio*** | **P-Value** |
| --- | --- | --- | --- |
| Activity-regulated cytoskeleton-associated protein | Inhba | 1,66369 | 0,0007495 |
| Neuronal pentraxin II | Stmn4 | -1,91215 | 4,73E-06 |
| Serum/glucocorticoid regulated kinase 1 | Opalin | -1,7873 | 0,0002542 |
| TAF15 RNA | Gabarapl1 | -1,5367 | 1,21E-07 |
